# Critical Care and Perioperative Monitoring

**DOI:** 10.1155/2014/737628

**Published:** 2014-05-04

**Authors:** Dimitrios Karakitsos, Mahmoud El Barbary, Lawrence Marshall Gillman, Apostolos Papalois, Ariel Shiloh

**Affiliations:** ^1^Department of Internal Medicine, University of South Carolina, School of Medicine, Columbia, SC, USA; ^2^Department of Anesthesiology, Division of Critical Care Medicine, Keck School of Medicine, The University of Southern California, Los Angeles, CA, USA; ^3^King Abdul-Aziz Cardiac Center, King Saud Bin Abdulaziz University for Health Sciences, Riyadh, Saudi Arabia; ^4^Department of Surgery, University of Manitoba, Z3053-409 Tache Avenue, Winnipeg, MB, Canada R2H 2A6; ^5^Critical Care Medicine Department, Athens University School of Nursing, 516 Mesogeion Avenue, Agia Paraskevi, 15342 Athens, Greece; ^6^Division of Critical Care Medicine, Department of Medicine, Jay B. Langner Critical Care Service Montefiore Medical Center, Albert Einstein College of Medicine, NY, USA

Advances in perioperative and critical care monitoring have greatly improved the standard of care during the last decades. However, no monitoring tool, no matter how accurate, by itself has improved critical care patients outcome [1]. Moreover, aside from lung-protective mechanical ventilation there has really been no consistent intervention that has individually resulted in improved outcomes.

The purpose of a monitoring system is not to treat but to provide clinical information that may impact medical decision-making. Various techniques have been implemented in the pre-, intra-, and postoperative monitoring of surgical patients. Invasive and noninvasive methods facilitate the monitoring of nervous, cardiovascular, respiratory, renal, and hematologic systems as well as of metabolic status. While monitoring will not prevent all adverse incidents in the perioperative period, it reduces the risks of accidents by permitting the continuous recording of core data such as heart rate, blood pressure, and peripheral oxygen saturation. Monitoring facilitates the detection of the consequences of human errors, while alerting physicians that a patient's condition is deteriorating for other reasons [2–6].

The prevention of perioperative complications has obvious implications both to patients and to health care systems. With over 230 million surgical procedures performed annually around the globe, the successful management of perioperative complications either in the operating room or in the intensive care unit (ICU) is becoming a major concern for health care providers. Interestingly, up to 4% of noncardiac surgery patients may die and more will develop postoperative complications that will prolong the duration of ICU hospitalization and reduce long-term survival. In major surgery, even in groups with a low mortality rate, the rate of postoperative complications is rather high [7–10]. We still fail to answer many critical questions. Should we admit more postsurgical patients to the ICU? Is this a prudent strategy that could improve patients' outcome or would such a policy dramatically increase hospitalization costs without affecting their long-term survival? Although no definitive solution to the aforementioned dilemma exists, the application of multipurpose perioperative monitoring might prove to be a prudent and cost-efficient strategy. Hence, this issue of the journal is presenting several articles outlining the important role of perioperative monitoring in modern clinical settings.

In recent years, a rather important development has been the gradual introduction of ultrasound technology in perioperative and critical care monitoring. The important role of this noninvasive, by-the-bed, and relatively cheap technology in the practice of modern anesthesiology and critical care is justified by the vast compendium of its applications in hemodynamic monitoring (echocardiography), neuromonitoring (transcranial color coded Doppler and ocular ultrasound), and guided procedures (vascular access and nerve blockade). Ultrasound has been introduced in medical school curriculums and resident training programs in several North American and European institutions. Our research group has recently presented the holistic approach (HOLA) concept of ultrasound imaging which defines critical care ultrasound as part of the patient examination by a clinician to visualize all or any parts of the body, tissues, organs, and systems in the patient`s life, anatomically and functionally interconnected state, and the context of the whole patient's clinical circumstances. The application of ultrasound technology as an adjunct to physical examination may indeed change the face of perioperative and critical care monitoring in the upcoming years [11].

Physical examination remains a matter of particular concern to the ICU environment since the former is deprived of several of its physical elements. Apart from the physical examination and critical care ultrasound issues raised above, advances in the interpretation of arterial blood gases and in cardiorespiratory care became evident in recent years. The integration of the Stewart-Figge approach in the routine interpretation of arterial blood gases is becoming increasingly popular. This approach, amongst other things, aids in evaluating the anion gap value while taking into account its dependence on the concentrations of the nonvolatile weak acids, which in turn has improved our understanding regarding metabolic acidosis [12]. Another important development in respiratory monitoring has been the introduction of the new Berlin definition of acute respiratory distress syndrome (ARDS) as the pertinent task force has categorized ARDS as mild, moderate, and severe, without excluding the presence of heart failure [13]. This improvement of the ARDS definition corresponds to a simple clinical truth that there are indeed mixed types of pulmonary edema. Moreover, the imminent fusion of lung ultrasound and echocardiographic applications into general chest ultrasound cardiorespiratory monitoring protocols could further enhance our understanding of the aforementioned mixed types of pulmonary edema.

Conventional invasive and noninvasive ventilation have been the mainstay of ARDS therapy in critical care settings. Recently, the role of extracorporeal membrane oxygenation (ECMO) has been upgraded in the management of severe respiratory and circulatory failure. ECMO has been brought out of the operating room and to the bedside allowing clinicians to aid in the care of critically ill patients requiring cardiac or cardiopulmonary support, but it has also become remarkably portable and thus allowed for intra- and interhospital transport of otherwise unstable patients. Venoarterial ECMO provides both respiratory and hemodynamic support, in contrast to venovenous ECMO, which provides only respiratory support. VA ECMO is ideally placed in a patient with a reversible pathological process and is commonly placed in those with cardiogenic shock from any number of etiologies including myocardial infarction, postcardiac surgery with the inability to wean off bypass, early graft failure following heart transplantation, and myocarditis. Other conditions for which VA ECMO may be considered include pulmonary embolism, septic or peripartum cardiomyopathy, or trauma to the great vessels. In the case of myocardial infarction leading to cardiac arrest, peripheral VA ECMO can provide hemodynamic stabilization until the neurologic status of the patient is determined—a therapeutic strategy called bridge-to-decision [14]. Although the efficacy of ECMO in improving long-term survival remains questionable, it is extremely useful when used to replace some of the function of a failed cardiopulmonary system and to provide some rest to the myocardium. Apart from the upgraded role of ECMO in modern cardiorespiratory care, lung-protective ventilation with the use of low tidal volumes and positive end expiratory pressure remains the standard of care in the ICU. Interestingly, the use of a lung-protective ventilation strategy in intermediate- and high-risk patients undergoing major abdominal surgery has been suggested to be associated with improved clinical outcomes and reduced health care utilization by the IMPROVE group [15].

Another fundamental parameter of perioperative monitoring is the evaluation of hemodynamic status. Hemodynamic monitoring and thus management have greatly developed in recent years. Technologies have evolved from invasive to noninvasive, and the philosophy has shifted from a static approach to a dynamic one. Ultrasound technology has indeed contributed much to the aforementioned shift in current monitoring strategies. The application of several other noninvasive technologies have equally contributed towards that direction. However, a breach still exists between clinical research studies evaluating noninvasive hemodynamic monitors and clinical practice. There are not yet enough data, especially in the perioperative period, to suggest that hemodynamic monitoring systems coupled with goal directed therapies could improve patient outcome [1]. We have recently had suggestions that therapy guided by the tried and true method of invasive hemodynamic monitoring via the pulmonary artery catheter may not be as sound as we previously thought. Due to great technological advances we have witnessed the introduction of multiple new monitoring devices over the last decade. However, we must be careful to view these new devices with a combination of both cautious optimism and slight uncertainty until their clinical utility can be proven. In the same way we must question the utility of existing devices rather than accepting the status quo and continuing their use based solely on historic pretenses.

Surely, the prevention of perioperative complications is of vital importance for anyone caring for this group of patients. Developing systems that can avoid the complications occurring in the first place and thereafter identifying and treating complications when they arise represent the basic logistics of modern perioperative monitoring. The physiological derangement of patients in the operating room and/or in the ICU has led to the development of sophisticated continuous monitoring systems. The prudent evaluation and application of the latter could in turn enable the prioritization of all available health care resources to individual cases. Notwithstanding, monitoring alerts physicians' senses and aids in guiding therapy but is not a therapy by itself.

***Dimitrios Karakitsos*** ***Dimitrios Karakitsos*** ***Mahmoud El Barbary*** ***Mahmoud El Barbary*** ***Lawrence Marshall Gillman*** ***Lawrence Marshall Gillman*** ***Apostolos Papalois*** ***Apostolos Papalois*** ***Ariel Shiloh*** ***Ariel Shiloh***
